# Construction of an Improved Air Quality Index: A Case Report

**Published:** 2019-08

**Authors:** Rui ZHAO, Yibo ZHANG, Sidai GUO

**Affiliations:** 1. Faculty of Geosciences and Environmental Engineering, Southwest Jiaotong University, Chengdu, 611756, China; 2. Sichuan Province Cyclic Economy Research Center, Southwest University of Science and Technology, Mianyang, 621010, China

**Keywords:** Air quality index, PM_2.5_, Health risk

## Abstract

We report a case to provide an improved air quality index (AQI) based upon association between individual health risk and Particulate matter (PM_2.5_) exposure. A Poisson sampling distribution model was used to quantify the health risk, in which the coefficient of exposure-response was derived from a simple Meta-analysis. The result shows that the old people are the most vulnerable population while exposing to PM_2.5_, for which they are advised to reduce intensity of their physical activities. It is expected that this study is insightful to create a nexus between air quality information and public communication, which help publics take appropriate actions on health protection.

## Introduction

Particulate matter with an aerodynamic diameter smaller than 2.5 micrograms (PM_2.5_) has a significant impact on human health (1). PM_2.5_ causes a rising mortality and morbidity of respiratory diseases (2), myocardial infarctions (3), cardiac arrhythmias (4) etc. In response to the precaution of PM2.5 associated healthy issues, Air Quality Index (AQI) provides information on real-time ambient air quality, to divide the degree of air pollution into a numerical scale, the larger the value, the greater the air pollution (5).

This brief communication proposes a method to construct a framework of improved AQI based upon individuals’ health risk resulted from exposure to PM_2.5_, to increase public awareness and take actions on protection of their physical health.

## Methods

Health risk assessment is a process to measure adverse impact on human health resulted from exposure to a toxicant, which is indicated by Possion sampling distribution, given as follows (6):

[1] $$\lambda_{i}(t)=k\cdot e^{\beta c}$$

where *λ* is the probability that human exposed to a specific pollutant, *i.e.* PM_2.5_ in the study, *t* the exposure period, *β* the coefficient of exposure-response, *c* the exposure concentration (μg/m^3^).

The coefficients of exposure-response for different receptors by considering their related health effects are derived from a simple Meta-analysis (inverse variance method) of the published epidemiological studies in China, given in Table 1. The overall coefficient for each receptor is an average of three coefficients, i.e. 0.76 %, 0.64 %, 0.74 %, for children, adult and old people, respectively.

**Table 1:** Exposure-response coefficient of different receptors

***Health effect***	***β /% (95% confidence interval)***		
	***Children***	***Adult***	***Old people***
Respiratory disease	0.86 (0.34∼0.97)	0.85 (0.24∼0.99)	0.70 (0.56∼1.07)
Hospital admission for respiratory disease	0.73 (0.45∼0.87)	0.69 (0.56∼0.89)	0.94 (0.84∼1.58)
Mortality due to respiratory disease	0.69 (0.23∼1.23)	0.38 (0.14∼1.27)	0.58 (0.44∼0.93)

The minimum PM _2.5_ concentration inhaled by a receptor is set as a reference concentration (*C*_0_), e.g. 25 *μ*g/*m*^3^, 50 *μ*g/*m*^3^, 35 *μ*g/*m*^3^ tolerated by children, adult and old people, respectively (7). Thus, the Eq. (1) is transformed as follows:

[2] $$\lambda_{0}(t)=k\cdot e^{\beta c_{0}}$$

Based on Eq. (1) and (2), the health relative risk (RR) is measured as follows:

[3] RR=λi(t)λ0(t)=eβ(C−c0)

Let the actual intake of PM_2.5_ within a certain time period is *m_inh_* (t), which can be expressed as follows:

[4] $$m_{inh}(t)=\int_{t_{2}}^{t_{1}}\eta_{1}cv\;dt$$

where *η*_1_ is the aspiration efficiency, that may be replaced by inhalable fraction of PM_2.5_, *t* the time, *c* the actual concentration of PM _2.5_, *v* the respiration rate.

Vincent (1990) (8) proposed an empirical expression to measure the inhalability *η*_1_ by using the particle aerodynamic diameter, given as follows:

[5] $$\eta_{1}=1-0.5\left\{1-{\left[7.6\times 10^{-4}{\left(d_{ae}\right)}^{2.8}+1\right]}^{-1}\right\}$$

where *d_ae_* is the particle aerodynamic diameter (*μ*g/*m*^3^).

Giorgini et al. (2016) (9) have indicated that individuals may have different respiration rate *v* under varying degree of activities, which are listed in Table 2.

**Table 2:** Respiration rate for different individuals under varying degree of activities

***Individuals***	***Moderate and light activities***	***Intense activities***
Children	1.2	1.9
Adult	1.6	3.2
Old people	1.6	3.2

By substituting the Eq. (4) into the Eq. (3), the relative risk is ultimately expressed as follows:

[6] $$\text{RR}=\text{exp}(\eta_{1}\beta \int_{t_{1}}^{t_{2}}v(c-c_{0})dt)$$

## Results

Table 3 shows the health relative risk of different individuals resulted from the moderate and intense activities. As the increase of PM_2.5_ exposure concentration, the relative risks of the three defined groups of individuals increase gradually. It is obvious that the RR resulted from the intense activities is higher than that from the moderate activities. Especially, the old people have the greatest health relative risk. By taking the classifications of AQI that has been implemented in China as an example, the corresponding relative risks for different individuals are given in Table 4. It is suggested taking appropriate actions on individual health protection, when the RR value is equal or greater than 1.5, *i.e.* the exposure concentration is 1.5 times to the reference concentration, indicating that the air quality is in slight pollution.

**Table 3:** Relative risk of different individuals exposed to varying concentrations of PM_2.5_

***Concentration of PM2.5***	***Children***		***Adult***		***Old people***	
	***Moderate and light activities***	***Intense activities***	***Moderate and light activities***	***Intense activities***	***Moderate and light activities***	***Intense activities***
25.0	1.00	1.00	0.78	0.60	0.89	0.79
35.0	1.09	1.15	0.86	0.74	1.00	1.00
45.0	1.20	1.33	0.95	0.90	1.12	1.22
55.0	1.31	1.53	1.05	1.11	1.26	1.49
65.0	1.43	1.76	1.16	1.35	1.42	1.82
75.0	1.56	2.03	1.29	1.65	1.59	2.23
85.0	1.71	2.34	1.42	2.02	1.79	2.72
95.0	1.87	2.70	1.57	2.47	2.01	3.32
105.0	2.05	3.11	1.74	3.02	2.26	4.06
115.0	2.24	3.58	1.92	3.70	2.53	4.95
125.0	2.45	4.13	2.13	4.52	2.85	6.05
135.0	2.68	4.76	2.35	5.53	3.20	7.39
145.0	2.93	5.48	2.60	6.76	3.59	9.03
155.0	3.20	6.32	2.87	8.26	4.04	11.02
165.0	3.50	7.28	3.18	10.10	4.53	13.46
175.0	3.83	8.39	3.51	12.35	5.09	16.44
185.0	4.19	9.67	3.89	15.11	5.72	20.09
195.0	4.58	11.14	4.30	18.47	6.43	24.53
205.0	5.01	12.84	4.75	22.58	7.22	29.96
215.0	5.48	14.79	5.26	27.62	8.11	36.60
225.0	6.00	17.05	5.81	33.77	9.11	44.70
235.0	6.56	19.64	6.43	41.29	10.23	54.60
245.0	7.17	22.64	7.11	50.49	11.49	66.69
255.0	7.84	26.09	7.86	61.73	12.91	81.45
265.0	8.58	30.06	8.69	75.49	14.50	99.48
275.0	9.38	34.64	9.61	92.30	16.29	121.51
285.0	10.26	39.92	10.62	112.86	18.30	148.41

**Table 4:** Relative risks for different individuals under diverse air quality conditions

***AQI grade***	***PM2.5 concentration μg/m^3^***	***Children***		***Adult***		***Old people***	
		**Moderate and light activities**	**Intense activities**	**Moderate and light activities**	**Intense activities**	**Moderate and light activities**	**Intense activities**
Excellent	0∼35	<1.09	<1.15	<0.86	<0.74	<1	<1
Good	36∼75	1.09∼1.56	1.15∼2.03	0.86∼1.29	0.74∼1.65	1∼1.59	1∼2.23
Light	76∼115	1.56∼2.24	2.03∼3.58	1.29∼1.92	1.65∼3.7	1.59∼2.53	2.23∼4.95
Moderate	116∼150	2.24∼3.20	3.58∼6.32	1.92∼2.87	3.7∼8.26	2.53∼4.04	4.95∼11.02
Serious	151∼250	3.20∼7.84	6.32∼26.09	2.87∼7.86	8.26∼61.73	4.04∼12.91	11.02∼81.45
Severe	251∼350	>7.84	>26.09	>7.86	>61.73	>12.91	>81.45

## Discussion

Most of the AQIs currently in use are simple in calculation by comparing each pollutant in the index to its standard (10). However, they are not able to reflect the concentration-response relationship, which characterizes the association between air pollutant and human health (11). Clearly, more work is required to develop an improved AQI with widespread appeal to the public health.

The association of AQI with health risk is insightful to create a nexus between air quality information and public communication. Prior studies have identified the vulnerable subpopulations related to exposure of air pollution, e.g. children, old people (12). The study further confirms that the elderly may be the most vulnerable population exposed to PM_2.5_. Thus, old people should reduce intensity of physical activities, especially on occasions where the concentration of PM_2.5_ exceeds the standard of air quality.

The major limitation of the study is the uncertainty for assessment of the health risk. First, the study takes PM_2.5_ as the representative air pollutant to demonstrate the construction of improved AQI. However, different air pollutants may have varying health endpoints, by which information may be incomplete through the use of a single indicator to reflect air quality (13). Secondly, the health relative risks are mainly derived from using local health statistics and air pollution standard. In such context, uncertainties are inherent in the quantitative measurement of exposure-response relationships. Because of data availability and validity, e.g. meteorological factors, individual immune mechanism etc. have been omitted. Besides, the prototype of improved AQI is impossible to provide specific health advice to individuals with health issues, instead of giving generic advice for each health risk category.

## Conclusion

The study employed the Poisson sampling distribution model to quantify health relative risk resulted from the PM_2.5_ exposure, in order to provide a framework of constructing an improved air quality index for China. It is expected that this approach is insightful in informing an improvement on the existing AQI system, to increase environmental health risk communication with publics, thus having potentials to take appropriate measures on individual health protection.

## Ethical considerations

Ethical issues (Including plagiarism, informed consent, misconduct, data fabrication and/or falsification, double publication and/or submission, redundancy, etc.) have been completely observed by the authors.
